# Phase I Clinical Trial of an Autologous Dendritic Cell Vaccine Against HER2 Shows Safety and Preliminary Clinical Efficacy

**DOI:** 10.3389/fonc.2021.789078

**Published:** 2021-12-16

**Authors:** Hoyoung M. Maeng, Brittni N. Moore, Hadi Bagheri, Seth M. Steinberg, Jon Inglefield, Kim Dunham, Wei-Zen Wei, John C. Morris, Masaki Terabe, Lee C. England, Brenda Roberson, Douglas Rosing, Vandana Sachdev, Svetlana D. Pack, Markku M. Miettinen, Frederic G. Barr, Louis M. Weiner, Sandhya Panch, David F. Stroncek, Lauren V. Wood, Jay A. Berzofsky

**Affiliations:** ^1^ Vaccine Branch, Center for Cancer Research, National Cancer Institute, Bethesda, MD, United States; ^2^ Radiology and Imaging Sciences, Clinical Center, National Institutes of Health, Bethesda, MD, United States; ^3^ Biostatistics and Data Management Section, National Cancer Institute, Rockville, MD, United States; ^4^ Clinical Support Laboratory, Applied/Developmental Research Directorate, Frederick National Laboratory, Frederick, MD, United States; ^5^ Department of Oncology, Karmanos Cancer Institute, Wayne State University, Detroit, MI, United States; ^6^ Division of Hematology-Oncology, University of Cincinnati, Cincinnati, OH, United States; ^7^ Cardiovascular Branch, National Heart, Lung, and Blood Institute, Bethesda, MD, United States; ^8^ Laboratory of Pathology, Center for Cancer Research, National Cancer Institute, Bethesda, MD, United States; ^9^ Department of Oncology, Georgetown Lombardi Comprehensive Cancer Center, Washington, DC, United States; ^10^ Center for Cellular Engineering, Clinical Center, National Institutes of Health, Bethesda, MD, United States

**Keywords:** cancer vaccine, HER2, immunotherapy, dendritic cell, clinical trial

## Abstract

**Background:**

Despite recent advances, there is an urgent need for agents targeting HER2-expressing cancers other than breast cancer. We report a phase I study (NCT01730118) of a dendritic cell (DC) vaccine targeting HER2 in patients with metastatic cancer or bladder cancer at high risk of relapse.

**Patients and Methods:**

Part 1 of the study enrolled patients with HER2-expressing metastatic cancer that had progressed after at least standard treatment and patients who underwent definitive treatment for invasive bladder cancer with no evidence of disease at the time of enrollment. Part 2 enrolled patients with HER2-expressing metastatic cancer who had progressed after anti-HER2 therapy. The DC vaccines were prepared from autologous monocytes and transduced with an adenoviral vector expressing the extracellular and transmembrane domains of HER2 (AdHER2). A total of five doses were planned, and adverse events were recorded in patients who received at least one dose. Objective response was evaluated by unidimensional immune-related response criteria every 8 weeks in patients who received at least two doses. Humoral and cellular immunogenicity were assessed in patients who received more than three doses.

**Results:**

A total of 33 patients were enrolled at four dose levels (5 × 10^6^, 10 × 10^6^, 20 × 10^6^, and 40 × 10^6^ DCs). Median follow-up duration was 36 weeks (4–124); 10 patients completed five doses. The main reason for going off-study was disease progression. The main adverse events attributable to the vaccine were injection-site reactions. No cardiac toxicity was noted. Seven of 21 evaluable patients (33.3%) demonstrated clinical benefit (1 complete response, 1 partial response, and 5 stable disease). After ≥3 doses, an antibody response was detected in 3 of 13 patients (23.1%), including patients with complete and partial responses. Lymphocytes from 10 of 11 patients (90.9%) showed induction of anti-HER2 responses measured by the production of at least one of interferon-gamma, granzyme B, or tumor necrosis factor-alpha, and there were multifunctional responses in 8 of 11 patients (72.7%).

**Conclusions:**

The AdHER2 DC vaccine showed evidence of immunogenicity and preliminary clinical benefit in patients with HER2-expressing cancers, along with an excellent safety profile. It shows promise for further clinical applications, especially in combination regimens.

## 1 Introduction

Human epidermal growth factor receptor 2 (HER2/Erb-B2) is overexpressed or amplified in multiple solid tumors. Homodimerization or heterodimerization of HER2 receptors with the same or other HER family members results in autophosphorylation of a tyrosine residue in the cytoplasmic domain, driving tumorigenesis (1–7).

The outlook for patients with HER2-positive breast cancer significantly improved in 1998 with FDA approval of trastuzumab and subsequent approval of several additional anti-HER2 agents. However, patients often show primary or secondary resistance to HER2 treatments, leading to relapse or disease progression. The extensively studied mechanisms of resistance include intrinsic, extrinsic, or combination mechanisms, including loss or alteration of the target or its downstream pathway (8, 9). Targeting one epitope with monoclonal antibodies eventually exerts pressure for immune escape or changes in signaling machinery, contributing to eventual loss of clinical efficacy. A further concern is that approximately 15% of patients receiving anti-HER2 regimens showed an increased risk of cardiac dysfunction (10, 11). Finally, FDA-approved indications for therapeutics targeting HER2 are limited to breast cancer and gastric cancer only, leaving an unmet need for many other HER2-expressing cancers (12–14).

Park et al. reported total regression of large established orthotopic HER2-expressing breast cancers up to 3,000 mm^3^ in the mouse TUBO model after the vaccination with a recombinant adenovirus (Ad) expressing the extracellular domain (ECD) and transmembrane domain (TMD) (ECTM) of rodent HER2. The viral vaccine also prevented autochthonous mammary carcinomas in HER2-transgenic mice, whether given as a free adenovirus or as syngeneic dendritic cells (DCs) transduced with adenovirus. The mechanism of protection involved antibody-mediated blockage of HER2 phosphorylation that was independent of Fc receptors (15–17). To expand the study for human HER2-expressing cancers, an Ad5f35 vector expressing the human HER2 extracellular (EC) and transmembrane (TM) domains (ECTM) (AdHER2) was generated. The Ad5f35 vector was used to better target DCs and reduce neutralization by pre-existing anti-Ad5 antibodies prevalent in the population. The vector modification changes the receptor tropism of the adenovirus from coxsackievirus and adenovirus receptor to CD46, which allows more efficient transduction in most human cells, especially DCs (18, 19). When AdHER2 was tested for anti-HER2 response in a syngeneic human HER2 transgenic mouse model, the immune sera inhibited HER2-positive SKBR3 cell survival in a dose-dependent manner. The mechanism of protection was through antibodies and was Fc receptor-independent, which differs from one of the major mechanisms of trastuzumab, which is Fc receptor-dependent (19–21). We translated these preclinical findings into a first-in-human phase I clinical trial to determine the safety and immunogenicity of an autologous DC vaccine transduced with AdHER2.

## 2 Patients and Methods

### 2.1 Patient Eligibility

Patients ≥18 years old with histologically confirmed HER2+ cancer were eligible for the study. Patients must have either recurrent or metastatic cancer that had progressed after at least one regimen of standard or adjuvant treatment. Patients with muscle-invasive bladder cancer must have completed curative resection and standard or neoadjuvant chemotherapy. Patients with metastatic disease were required to have at least one target lesion, as defined by Response Evaluation Criteria in Solid Tumors (RECIST) v.1.1. Additional eligibility criteria included ECOG performance status of 0 or 1, NYHA heart failure class I, left ventricular ejection fraction ≥53% by echocardiogram, and adequate organ function for bone marrow (absolute neutrophil count ≥ 1,000 cells/mm^3^ and absolute lymphocyte count ≥ 300 cells/mm^3^), kidney (creatinine ≤ 1.5 mg/dl), and liver (SGOT and SGPT ≤3 times the upper limits of normal and total bilirubin ≤1.5 mg/dl). Exclusion criteria included pregnancy, active brain metastasis, cumulative dose of doxorubicin ≥400 mg/m or epirubicin ≥800 mg/m^2^, medical conditions requiring systemic corticosteroids, and autoimmune condition requiring active intervention or active infection requiring treatment. No concurrent cancer treatment was allowed except for ongoing hormone therapy for breast cancer.

### 2.2 Clinical Trial Design and Regulatory Oversight

**Figure 1:**
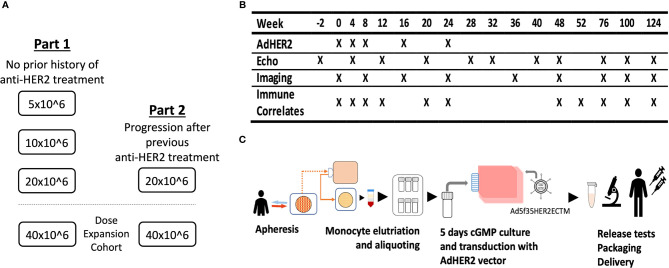
The clinical trial design of the AdHER2 DC vaccine. **(A)** Part 1 with dose escalation was opened to enroll 1) patients with metastatic cancer that progressed after at least one standard therapy or 2) with high-risk bladder cancer who completed treatment with curative intent and no radiographic evidence of disease. After reviewing the safety data of part 1, part 2 and dose expansion cohorts followed. **(B)** Study calendar showing schedules for vaccination and assessments. **(C)** Diagram showing AdHER2 DC vaccine manufacturing. Briefly, mononuclear cells of patients were collected by apheresis and elutriated monocyte aliquots were stored frozen until each vaccine dose was manufactured. On day 0, one aliquot was thawed and resuspended in media containing cytokine and plasma. On day 2, the medium was changed and keyhole limpet hemocyanin was added as an immune adjuvant. On day 3, cells were transduced with the AdHER2 vector designed to express the ECTM of HER2. Then, the maturation cocktail was added. On day 4, the product was reviewed and packaged for administration.

This phase I dose-escalation clinical trial was designed to demonstrate the safety and immunogenicity of an autologous AdHER2 DC vaccine in patients with HER2-expressing cancer. The study included two cohorts. Part 1 with dose escalation [5 × 10^6^, 10 × 10^6^, and 20 × 10^6^ DCs per dose, six patients per dose level (DL)] was opened first in patients who were naive to any anti-HER2 therapy for HER2-expressing metastatic cancer or patients with bladder cancer that was surgically treated with curative intent with or without standard-of-care (neo)adjuvant therapy with no evidence of disease at enrollment. Part 2 (DL 20 × 10^6^) enrolled patients who had progressed after HER2-targeted therapy. Parts 1 and 2 also opened dose-expansion cohorts (DL 40 × 10^6^) (**Figure 1**). The primary endpoints of the study were to determine whether the vaccine was associated with cancer therapeutics-related cardiac dysfunction (CTRCD) and to determine the immunogenicity of the vaccine against HER2.

The study was approved by the Institutional Review Board of the National Cancer Institute (NCI), National Institutes of Health (NIH) and conducted in accordance with institutional and federal guidelines for human investigation in accordance with the Declaration of Helsinki. All participants were informed of the investigational nature of the study and provided written informed consent prior to enrollment. This trial was sponsored by the Center for Cancer Research of the NCI. Ongoing safety oversight was conducted by the Institutional Review Board and Safety Monitoring Committee at the NIH. Any serious adverse events (AEs) were reported to the U.S. Food and Drug Administration.

### 2.3 AdHER2 DC Vaccine Vector Construction, Vaccine Manufacturing, and Administrations

The adenoviral construct AdHER2 was developed by the Baylor Center for Cellular and Gene Therapy following Good Manufacturing Practice regulations. It was constructed by inserting the ECTM sequence of human HER2 into the E1a/E1b region of the Ad5 vector as a backbone; the Ad35 knob and fiber substituted for the corresponding Ad5 regions. The intracellular domain (ICD) of HER2 was not included to avoid any potential of signal transduction and oncogenicity. The product is an Ad5f35 vector expressing the ECTM domains of human HER2.

The autologous DC vaccine was manufactured at the Center for Cellular Engineering at the NIH Clinical Center (**Figure 1**) (22). The mononuclear cells of the patients were collected by apheresis and elutriated monocyte aliquots were stored frozen until each vaccine dose was manufactured, targeting up to 8 aliquots of 3.33 × 10^8^ cells each.

On day 0, one cryopreserved monocyte-enriched aliquot was thawed and resuspended in media containing 90% RPMI-1640; 10% autologous heat-inactivated filtered plasma or allogeneic heat-inactivated, irradiated, filtered AB plasma; 2,000 IU/ml rGM-CSF (sargramostim, Genzyme, Cambridge, MA, USA); 10 μg/ml gentamicin; and 2,000 IU/ml USP grade recombinant human IL-4 (CellGenix, GmbH, Freiburg, Germany) at a final concentration of 1.5 × 10^6^ cells/ml in T75 flasks (Corning Incorporated Life Sciences, Lowell, MA, USA). The flask was incubated at 37°C in 5% CO_2_. On day 2, the medium was changed and keyhole limpet hemocyanin (KLH, 10 μg/ml, Stellar Biotechnologies, Inc., Port Hueneme, CA, USA) was added to the final concentration, 10 μg/ml. On day 3, a 24-h sterility test sample was collected from the immature DCs in the flask, followed by transduction with the AdHER2 vector designed to express the ECTM of HER2 at a ratio of 3,000 viral particles per cell plated. The maturation cocktail consisting of LPS (30 ng/ml, List Laboratories, Inc., Campbell, CA, USA) and IFN-γ (1,000 IU/ml, Horizon Therapeutics, Dublin, Ireland) was added at 20 h before harvesting. On day 4, the product was reviewed for the release criteria, including cell count, viability, CD83, CD340 (HER2/neu), gram stain, endotoxin, and 24-h sterility (day 3 specimen obtained prior to the transduction). The vaccine product was packaged with infusion media in a total volume of 1 ml Plasma-Lyte A (Baxter, Deerfield, IL, USA) containing 10% autologous heat-inactivated plasma or allogeneic heat-inactivated, filtered AB plasma in a sterile syringe, and the expiration was set at 4 h after packaging. A final certificate of analysis including bacterial and fungal culture (14 days) and mycoplasma test by PCR was issued for each dose.

Five vaccine doses were scheduled at weeks 0, 4, 8, 16, and 24 (**Figure 1**). The vaccine was administered intradermally in two sites, 0.5 ml each, in alternating arms starting with the non-dominant arm. Patients were monitored for vital signs at baseline and every 15 min for 60 min after the first vaccination and at baseline and at 15 min in subsequent doses. Vaccine report cards were provided to record injection-site reactions. Patients who demonstrated disease progression during the vaccine manufacturing did not receive the vaccine. Selected patients who were medically stable continued to receive vaccine doses as scheduled at initial documentation of progression while they were transitioning to their care in coordination with their primary oncologist.

### 2.4 Adverse Events and Dose-Limiting Toxicity

Patients were evaluated by physical exam and laboratory tests every 4 weeks until progression or week 40 and every 8–12 weeks thereafter until week 124. Patients were evaluated with echocardiogram at baseline and on weeks 4, 12, 20, 28, 32, 40, 48, 76, 100, and 124 if no abnormalities were observed in a prior echocardiogram. All patients who received at least one dose of the vaccine were evaluated for safety. AEs were reported following the revised NCI Common Terminology Criteria for Adverse Events version 4.0. The study-defined dose-limiting toxicities (DLTs) were grade ≥2 allergic reactions, grade ≥2 autoimmune disorder, grade ≥3 cardiac disorders or injection-site reactions, grade ≥3 anaphylaxis anytime during the study, and grade ≥3 dermatologic, gastrointestinal, renal, urinary, hepatic, or neurologic toxicity within 30 days after the vaccination that were at least possibly related to the vaccine. Study-defined immunization-related DLTs included grade ≥3 anaphylaxis and injection-site reactions. The clinical data cutoff date was November 1, 2019.

### 2.5 Objective Response Evaluation

All patients available at the first objective response evaluation at week 8 and who received at least two doses of the vaccine were deemed evaluable. Restaging CT or MRI scans were done every 8 weeks in the first 12 months then every 12–24 weeks until progressive disease (PD) or any clinical event requiring imaging studies. Bone scan was obtained when clinically indicated. Objective responses were evaluated by modified immune-related response criteria (irRC) with unidimensional measurement of target lesions (23). Patients who showed clinical benefit [complete response (CR), partial response (PR), or stable disease (SD)] were compared with patients who had no clinical benefit. All scans were assessed by clinical radiologists, then reviewed by an independent radiologist who specializes in response evaluation in clinical trials at the NIH Clinical Center.

### 2.6 Immune Correlatives

Samples from patients who received at least three doses of the vaccine were evaluated for immunogenicity. Immune correlatives were evaluated on weeks 0, 8, 16, 24, 32, 40, 48, 60, 76, 100, and 124. If the condition of the patient was not feasible for the research biospecimen collection, IRB-reported deviated timepoint samples were used in limited occasions. Sera and peripheral blood mononuclear cells (PBMCs) were stored frozen until the time of analysis.

#### 2.6.1 HER2-Specific Antibody Response

Serum collections were analyzed for HER2 peptide-specific antibody response using a panel of overlapping 15-mer peptides spanning the HER2 sequence using PepStar peptide microarrays, Multiwell Microarray Service (JPT Peptide Technologies GmbH, Germany). The reactivity pattern was summarized in fold increase compared with baseline. As this is a study conducted in non-HLA preselected patients with a small number of available biospecimens, responses to multiple epitopes in individual patients or detection of varying epitopes among patients will be reported in a descriptive manner.

#### 2.6.2 HER2-Specific Cellular Response

Assays were validated and performed following standard operating protocols at the Clinical Support Laboratory, Leidos Biomedical Research, Inc., Frederick National Laboratory, Frederick, MD, USA.

##### 2.6.2.1 Cell Culture Conditions

PBMCs were obtained by density gradient centrifugation using Ficoll-Hypaque (GE Healthcare, Chicago, IL, USA) and stored frozen until the testing of multiple timepoints to avoid interassay variability. Frozen PBMCs were thawed, resuspended, and plated into 24-well tissue culture plates (Corning, Corning, NY, USA) at 2–3 × 10^6^ viable cells/well in CTL media containing RPMI (Thermo Fisher Scientific, Waltham, MA, USA), 10% human AB serum (Omega Scientific, Inc., Tarzana, CA, USA), and 1% Pen/Strep-L-glutamine (Gibco, Thermo Fisher Scientific, Waltham, MA, USA). These PBMCs were stimulated with either the HER2 ECD or the HER2 ICD peptide mix (PM-ERB_ECD, PM-ERB_ICD; JPT Peptide Technologies GmbH, Germany) at 1 µg/ml in a 37°C humidified 5% CO_2_ atmosphere in the presence of recombinant human IL-7 (5 ng/ml, Peprotech, Cranbury, NJ, USA) on day 0. A healthy donor control was stimulated in a similar way with 1 μg/ml CMVpp65 peptide (Mimotopes Pty Inc., Australia). Recombinant human IL-2 (Tecin; Hoffmann-La Roche, Switzerland) was added on day 3 at 12.5 units/ml. Cells were fed every 2–3 days by removing half of the culture supernatant and replacing it with fresh CTL media containing IL-7 and IL-2. The cells were harvested on days 10–12. If the cultures needed to be fed <48 h before the assay, the media were replaced without the addition of cytokines.

##### 2.6.2.2 FluoroSpot/ELISpot Assay

FluoroSpot and ELISpot assays were validated and performed following standard operating protocols at the Clinical Support Laboratory, Leidos Biomedical Research, Inc. All assays using PBMCs (100,000/well) were performed after *in-vitro*-stimulation for 10-12 days as the effectors and peptide-pulsed autologous DCs (10,000/well) as the antigen-presenting cells (APCs) at a 10:1 ratio in culture media containing RPMI (Gibco, Thermo Fisher Scientific, Waltham, MA, USA), 5% human AB serum (Omega Scientific), and 1% Pen/Strep-L-glutamine (Gibco, Thermo Fisher Scientific, Waltham, MA, USA). The APCs were pulsed with 1 µg/ml peptide 2 h at 37°C before being plated with the effectors. The response to the HER2 peptides and the control peptide HTLV-I (Tax 11–19, Mimotopes Pty Inc.), plus mitogenic stimulation with PHA, was assessed. For the IL-4 ELISpot, a 1:67 dilution of IL-4-I antibody (final 15 mg/ml, Mabtech, Inc. Cincinnati, OH, USA) was added to MSIP opaque plates (MilliporeSigma, Burlington, MA, USA) and incubated overnight at 4°C. The FluoroSpot plates have 3 pre-coated markers (monoclonal antibodies 1-D1K, GB10, and MT25C5) situated in 96-well low autofluorescent polyvinylidene fluoride (PVDF) membrane, HTS opaque plates (Mabtech, Inc.). On the day of assay, both the three-marker plates and the IL-4 plates were washed four or five times in D-PBS and blocked with 5% human AB culture medium at room temperature for approximately 2 h. The effectors and APCs were incubated for 18–20 h (three-marker plate) or 24 h (IL-4 plate) at 37°C and 5% CO_2_. The next day, the plates were manually washed five times with D-PBS, followed by a 2-h incubation at room temperature with a 1:200 dilution of anti-IFN-γ monoclonal antibody 7-B6-1-BAM, a 1:500 dilution of biotinylated anti-granzyme B monoclonal antibody GB11, a 1:200 dilution of anti-TNF-α monoclonal antibody MT20D9-WASP (three-marker plate), and a 1:1,000 dilution of IL-4-II-biotin antibody (Mabtech, Inc.) in D-PBS containing 0.5%–1% bovine serum albumin. After incubation and five washes in D-PBS to remove excess antibody, a 1:200 dilution of anti-BAM-490, SA-550, and anti-WASP-640 (Mabtech, Inc.) in D-PBS containing 1% bovine serum albumin was added to each well for 1 h at room temperature followed by five manual washes in D-PBS. IL-4 plates were treated with streptavidin-alkaline phosphatase (1:1,000 dilution, Mabtech Inc.) in D-PBS containing 0.5% fetal bovine serum. Finally, fluorescence enhancer (Mabtech, Inc.), 50 µL/well (three-color plates), or filtered BCIP/NBT phosphatase substrate (KPL), 100 µL/well (IL-4 plates), was added for 15 min, resulting in the development of spots. Plates were flicked to remove the enhancer (three-color plates) or washed three times with sterile water (IL-4 plates), then dried overnight in the dark with the underdrain removed. The spots were visualized and counted using the ImmunoSpot S6 Imaging Analyzer system (Cellular Technology Ltd., Cleveland, OH, USA) equipped with three separate filters and ImmunoSpot Fluoro-X software (Cellular Technology Ltd.) which utilizes individual monochromatic images taken at each excitation/emission condition optimized for each fluorochrome. These monochromatic images were then assessed by the software using an experimentally validated Center of Mass Distance algorithm to determine multicolor spot counts. The blue spots on the IL-4 plates were analyzed using ImmunoSpot software (Cellular Technology Ltd.). All wells were counted with set parameters and each count was verified to ensure the accuracy of the counting software. All results were expressed as the number of spots per 10^6^ responder cells after subtracting background spots obtained in wells of effectors with non-pulsed DCs. The results were reported as positive if the mean of the test specimen was greater than the control mean + 1 standard deviation (24). If there was a response in at least one of IFN-γ, granzyme B, or TNF-α, it was considered as having a positive post-vaccination response. Regarding multifunctional lymphocyte responses, the detection of simultaneous production in at least two of IFN-γ, granzyme B, or TNF-α in combination was considered as a positive response.

### 2.7 Statistical Analysis

An exact Wilcoxon rank sum test was used to compare previous lines of chemotherapy with and without clinical benefit. The same test was used to compare continuous measures such as baseline values of total white blood cell count, peripheral blood lymphocyte percentage and counts, lymphocyte subsets, IgG level, and 25-(OH)-vitamin D among the clinical benefit groups: clinical benefit (CB) and no clinical benefit (NCB). Several parameters were also evaluated relative to normal vs. low levels, and these were compared between the clinical benefit groups using Fisher’s exact test.

## 3 Results

### 3.1 Patient Characteristics

Between March 2013 and August 2019, 33 patients were enrolled in the study. Median follow-up was 36 weeks (range 4–124). Four patients completed the entire study, including safety visits up to 124 weeks from enrollment. All patients were off study at the time of data cutoff (**Table 1**). Females (*n* = 20) constituted 60.6% of enrolled patients, with a median age of 60 (range, 36–72). Of 30 patients with metastasis at the time of enrollment, 18 (60%) developed *de novo* metastatic disease and had an average of three treatment regimens prior to enrollment. No one was enrolled based on HER2 fluorescence *in situ* hybridization (FISH) with IHC 0. Patients in part 1 did not have any history of HER2-targeted therapy prior to enrollment. All patients in part 2 (*n* = 9) had received anti-HER2-containing regimens prior to enrollment. All patients had received trastuzumab and one patient (patient 32) had received trastuzumab and lapatinib prior to enrollment. Median cardiac left ventricular ejection fraction at time of enrollment was 63%.

**Table 1:** Patient characteristics.

		Part 1				Part 2		Total
		5 × 10^6^	10 × 10^6^	20 × 10^6^	40 × 10^6^	20 × 10^6^	40 × 10^6^	
Age	30–59	4	4	2	1	2	2	15
	≥60	3	4	4	2	4	1	18
Sex	Male	3	2	3	2	1	2	13
	Female	4	6	3	1	5	1	20
Race	African American		1					1
	Asian		2			1	1	4
	Caucasian	7	5	5	3	5	2	27
	Hispanic			1				1
Primary site of cancer	Breast					6	1	7
	NSCLC	1						1
	Esophageal/EGJ/stomach		1				1	2
	Colon/rectal	2	5	1			1	9
	Ovary	1	2	2				5
	Prostate			1	1			2
	Bladder, NED^a^			2	1			3
	Bladder, metastatic	2						2
	Uterine cervix	1			1			2
Previous lines of treatment^b^	0–2	2	3	4	3	2	3	17
	3 or more	5	5	2	0	4	0	16
Total		7	8	6	3	6	3	33

All patients were ECOG 0 or 1 by eligibility criteria. Part I: n = 24 (no previous HER2-targeted therapy). Part II: n = 9 (previously progressed after one or more HER2-targeted therapy).

a NED, no evaluable disease; vaccines were given as an adjuvant after the standard care.

b Number of treatment regimens prior to enrollment, excluding neoadjuvant or adjuvant regimens.

### 3.2 Toxicity

The AdHER2 DC vaccine was well tolerated. No allergic reaction, autoimmune or cardiac disorder, grade ≥3 injection-site reaction, or anaphylaxis was reported during the study. All the other AEs that fit the study-defined DLT criteria were more likely associated with disease progression than vaccination. No study-defined immunization-related DLT was reported. Twenty-two patients died during the study period, all associated with disease progression, not with vaccination. Of 47 grade ≥3 toxicities, 11 were at least possibly attributable to the investigational drug, although the clinical picture favored the effects of disease progression rather than vaccine administration or inflammatory response following vaccination (see **Table 2** for specific AEs). All but two patients had grade 1–2 injection-site reactions with each dose, beginning several hours after injection and resolving spontaneously in 4–5 days. Repeated doses did not aggravate injection-site reactions. Systemic AEs such as fever, chills, or myalgia were not suggestive of systemic inflammation; however, anemia, fatigue, and pain were associated with the course of underlying disease. CTRCD or heart failure was not observed in any patients during follow-up (**Table 2**).

**Table 2:** Reported adverse events (AEs) that were present in more than 10% (*n* = 4) of patients who received at least one dose of the vaccine (*n* = 31).

Toxicity		Number of patients	(%)
Hematologic disorders	Anemia	10	32
	White blood cell count decrease	4	13
	Lymphocyte decrease	15	48
Gastrointestinal disorders	Abdominal pain	11	36
	Ascites	6	19
	Bloating	5	16
	Diarrhea	7	23
	Dyspepsia	5	16
	Nausea/vomiting	8/10	26/32
	AST/ALT elevation	5	16
	Bilirubin/ALP elevation	5/6	16/19
General disorders and injection-site reactions	Fatigue	18	58
	Pain	10	32
	Injection-site reaction	29	94
Infections	Urinary tract infection	7	23
	Respiratory tract infection	7	23
Metabolism and nutritional disorders	Anorexia	8	26
	Weight loss	6	19
	Dehydration	5	16
	Hypoalbuminemia	7	23
	Hypophosphatemia	9	19
Musculoskeletal and connective tissue disorders	Back pain/flank pain	9/4	29/13
	Chest wall pain	4	13
Neoplasms	Tumor pain	4	13
Psychiatric disorders	Insomnia	4	13
Renal disorders	Creatinine elevation	7	23
Respiratory, thoracic, and mediastinal disorders	Cough	10	32
	Dyspnea	10	32
	Pleural effusion	4	13
Skin abnormality	Pruritus	3	10

Twenty-three patients (74%) had grade ≥3 AEs. Six patients (19%) had grade ≥3 AEs attributable at least possibly to vaccine.

### 3.3 Clinical Activity and Patient Course

Ten patients (30.3%) completed all five scheduled doses. Two patients were enrolled who did not receive the vaccine due to disease progression while waiting for the first dose. Twenty-six patients (78.8%) received the ≥3 doses required for assessment of immunogenicity. Reasons for stopping treatment included disease progression (*n* = 12), death (*n* = 5), development of medical conditions unsuitable to travel to the study site (*n* = 4), and patient request (*n* = 2).

**Figure 2:**
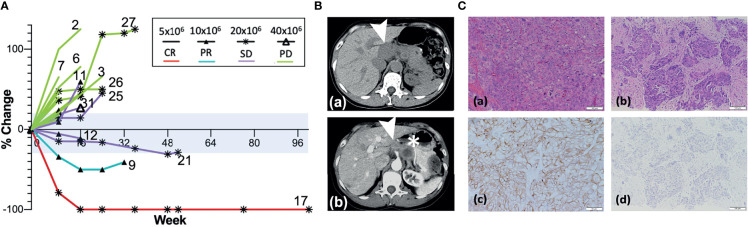
Responses after AdHER2 DC vaccination. **(A)** Best responses in evaluable patients. Note the durable response in patients 21 and 17 with non-target lesion progressions at the time of progression while their target lesions were still showing responses. Some patients were determined as having PD because non-target lesion progression was noted as shown in patient 12 who showed SD initially at week 8 but determined as PD at week 16 despite original target lesions remained in the range of SD. The range for a stable disease (SD, −30% to +20% change in the sum of target lesions) is tinted with light blue. Not all patients who progressed at week 8 were labeled with the patient number in the figure. The summary of the response type and duration can be found in **Table 3**. **(B)** CT scans of patient 9 at baseline **(a)** and at week 24 **(b)**. The target lesion (➤, 4.7 × 1.9 cm) at baseline decreased to 1.9 × 1.9 cm at week 24, but a non-target lesion (**∗**) progressed. **(C)** Microscopic exam of tumor tissue (×400) from patient 17; **(a, b)** H–E staining; **(c, d)** IHC of HER2; **(a, c)** oophorectomy specimen at the time of diagnosis; **(b, d)** at the time of recurrence showing high-grade serous ovarian cancer with HER2 3+ at diagnosis. **(d)** IHC of HER2 showing the absence of HER2 expression at the time of recurrence is suggestive of immune escape.

Among all patients available at the first objective response evaluation at week 8 and who received at least two doses of the vaccine, 21 patients were deemed evaluable for objective response (63.6% of 33 enrolled; 75% of 28 who received ≥2 doses); 7 patients (33.3% of evaluable patients) showed CB (CR = 1, PR = 1, and SD = 5) (**Table 3** and **Figure 2A**). Seven patients were determined inevaluable either because their scans or target lesions were considered inadequate per review by the independent radiologist (patients 1, 19, 22, 28, and 30), or because they had no evaluable disease by enrollment criteria for the bladder adjuvant treatment indication (patients 18 and 20). All patients with CB received 10 × 10^6^ or more DCs, whereas none of the five evaluable patients at the lowest DL of 5 × 10^6^ DCs showed clinical benefit. Patient 9 (DL 10 × 10^6^) with metastatic stomach cancer (HER2 IHC 3+, FISH 2.5) had a 50% decrease in the sum of target lesions at week 16 but progressed due to non-target lesion progression at week 24 (**Figure 2B**). Among three patients who showed a decreased sum of target lesions, two with ovarian cancer of different pathologic subtypes remained stable for 6 months or more. One patient (patient 17, DL 20 × 10^6^) with high-grade serous ovarian cancer (HER2 IHC 3+, FISH 1.3) had lesions confined to the vaginal cuff at the time of enrollment that completely regressed after four doses of vaccine. This response was first demonstrated at week 24 and lasted until week 113. The patient experienced episodes of small bowel obstruction requiring adhesiolysis (week 26), but no omental seeding was found, and repeated cytology of peritoneal fluid was also negative for malignant cells. Later, she had a surge in CA-125 past week 100, and CT scan confirmed recurrence with omental nodules. Biopsy of one of the omental lesions turned out to be HER2 IHC 0 FISH 1.2, suggestive of a possible escape variant (**Figure 2C**). A patient (patient 21, DL 20 × 10^6^) with carcinosarcoma-type ovarian cancer (HER2 1+, FISH 1.0) who enrolled after five lines of prior cancer treatment had a maximum 24.8% decrease in the sum of target lesions that lasted until week 48. Additional SD was observed in two patients with colorectal cancer (part I, DL 20 × 10^6^), one with male breast cancer (part 2, DL 40 × 10^6^), and one with cancer of the esophagogastric junction (part 2, DL 40 × 10^6^).

**Table 3:** Summary of objective responses: CR, complete response; PR, partial response; SD, stable disease; PD, progressive disease; NE, not evaluable; NED, no evaluable disease (bladder cancer adjuvant); N/A, not applicable.

Patient	Primary site	Dose levels	HER2		Vaccine doses^a^	Objective response	Response duration (weeks)
			IHC	FISH			
Part 1							
1	Colon	5 × 10^6^	2	1.6	4	NE	N/A
2	Ovarian	5 × 10^6^	2	1.2	3	PD	–
3	Cervix	5 × 10^6^	2	1.6	4	PD	–
4	NSCLC	5 × 10^6^	2	1.2	3	PD	–
5	Bladder	5 × 10^6^	3	4.8	0	NE	N/A
6	Bladder	5 × 10^6^	3	0.9	4	PD	–
7	Colon	5 × 10^6^	1	0.9	2	PD	–
8	Colon	10 × 10^6^	3	11.2	3	PD	–
9	Gastric	10 × 10^6^	3	2.5	5	PR (week 8)	16
10	Colon	10 × 10^6^	2	–	0	NE	N/A
11	Colon	10 × 10^6^	2	1.6	5	SD	16
12	Colon	10 × 10^6^	1	1.4	4	SD	16
13	Ovarian	10 × 10^6^	2	1	3	PD	–
14	Colon	10 × 10^6^	3	1.3	4	PD	–
15	Ovarian	10 × 10^6^	2	1.1	3	PD	–
16	Colon	20 × 10^6^	1	–	3	PD	–
17	Ovarian^b^	20 × 10^6^	3	1.3	5	CR (week 24)	89
18	Bladder	20 × 10^6^	3	1.3	5	NE (NED)	N/A
19	Prostate	20 × 10^6^	3	1.0	4	NE	N/A
20	Bladder	20 × 10^6^	1	1.2	5	NE (NED)	N/A
21	Ovarian^c^	20 × 10^6^	1	1.0	5	SD	48
22	Uterine cervix	40 × 10^6^	2	1.4	4	NE	N/A
23	Bladder	40 × 10^6^	1	1.2	1	NE (NED)	N/A
24	Prostate	40 × 10^6^	1	1.3	3	PD	–
Part 2							
25	Breast	20 × 10^6^	2	1.1	5	SD	24
26	Breast	20 × 10^6^	3	1.2	5	PD	–
27	Breast	20 × 10^6^	3	13.7	5	PD	–
28	Breast	20 × 10^6^	3	3.6	4	NE	N/A
29	Breast	20 × 10^6^	3	1.7	4	PD	–
30	Breast	20 × 10^6^	3	2.9	2	NE	N/A
31	EGJ^d^	40 × 10^6^	3	3.5	5	SD	24
32	Rectal	40 × 10^6^	2	–	1	NE	N/A
33	Breast	40 × 10^6^	2	–	1	NE	N/A

a Patients who received at least two doses were determined evaluable.

b High-grade serous ovarian cancer.

c Ovarian carcinosarcoma.

d Cancer of esophagogastric junction.

The number of previous lines of treatments did not differ between CB and NCB (CB: median 3, range 1–5 vs. NCB: median 2, range 1–4, *p* = 0.95). Patients with CB were found to have lower total lymphocyte percentage (*p* = 0.054; CB: median 15.5, range 10.3%–29.0%; NCB: median 25.25, range 7.8%–60.7%), absolute lymphocyte count (*p* = 0.083; CB: median 1,170, range 680–1,490/µl; NCB: median 1,460, range 500–2,530/µl), and absolute CD4 count (*p* = 0.031; CB: median 558, range 182–609/µl; NCB: median 754, range 244–1461/µl) at baseline compared with patients who progressed while on treatment. Baseline laboratory values including WBC, CD3, and CD8 T cell counts; IgG; and 25-(OH)-vitamin D level did not show any difference between CB and NCB.

Anti-adenoviral IgG was measured to evaluate the association of pre-existing adenoviral antibody that can neutralize adenovirus. Of 23 patients (CR = 1, PR = 1, SD = 4, PD = 9, not evaluable = 8) tested for adenovirus-specific IgG titer at baseline, 3 (13.0%) had baseline positivity (1:64, *n* = 2, both patients were not evaluable; 1:8, *n* = 1, SD). Results were available for both baseline and follow-up titer in 19 patients. Among those, seven (38.9%) who were negative at baseline were found to develop anti-adenoviral IgG titers suggestive of past infection (1:8 to 1:32, *n* = 5: 1 CR, 1 SD, 2 PD, and 1 not evaluable) or active infection (1:64, *n* = 2: 1 SD and 1 PD).

Among the unevaluable patients, three had muscle-invasive bladder cancer. They were enrolled as an adjuvant indication after completing standard-of-care (neo)adjuvant chemotherapy and curative resection. They did not have any measurable disease at enrollment. Two patients completed all scheduled doses; disease did not recur for >3 years. The third patient was unexpectedly taken off study after receiving the first dose due to an incidental finding of esophageal cancer (HER2 IHC 0) not associated with the vaccine or bladder cancer.

### 3.4 Immune Responses

Limited numbers of paired baseline and post-vaccination samples were available for evaluation for humoral (*n* = 13) and cellular (*n* = 11) immunogenicity.

#### 3.4.1 Antibody Response Against HER2 by Peptide Array

**Figure 3:**
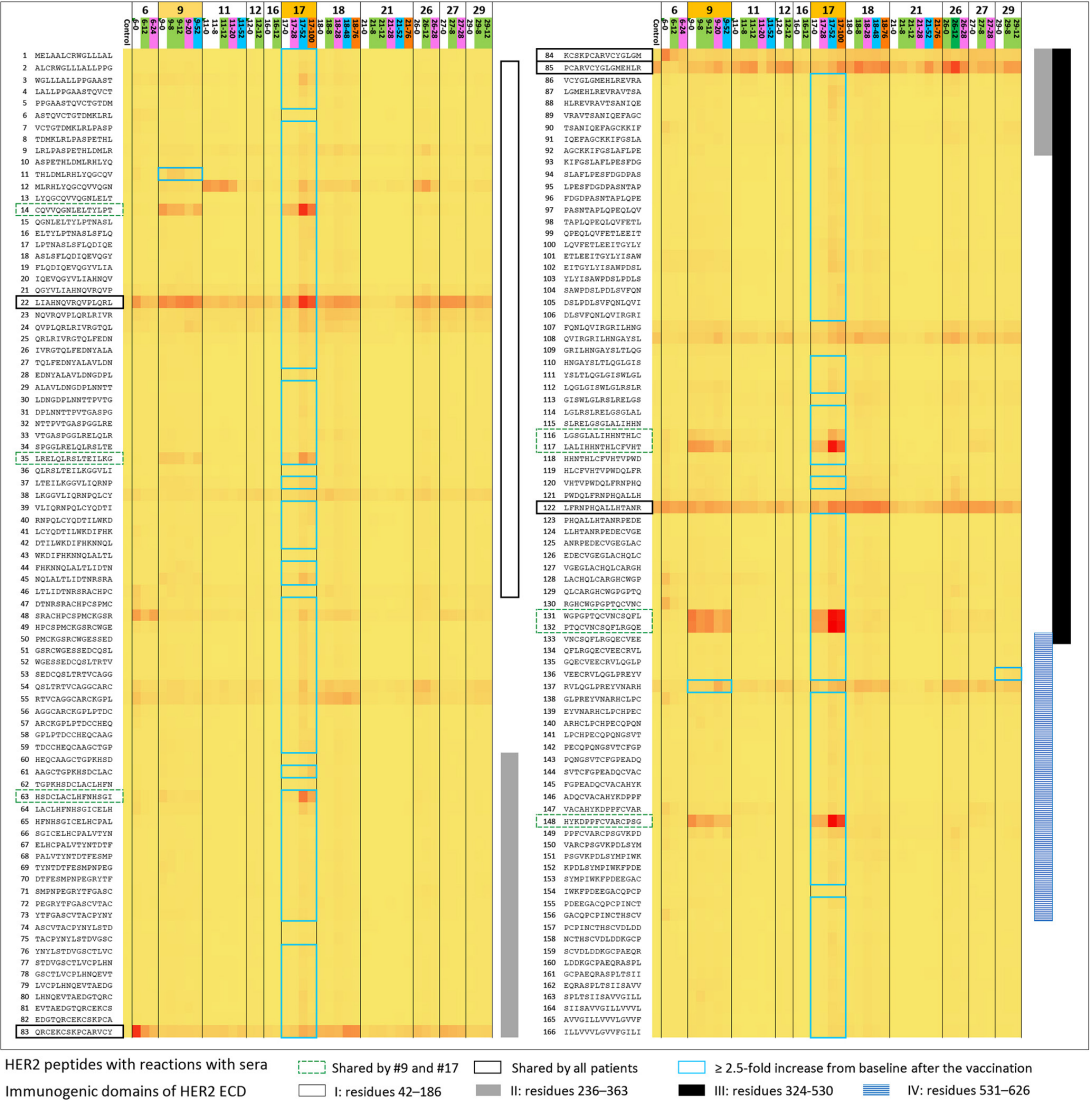
Antibody response against extracellular domain (ECD) and transmembrane domain (TMD) of HER2 after vaccination using peptide array (from left to right; patients 6, 9, 11, 12, 16, 17, 18, 21, 26, 27, and 29 with the two best responders—patients 9 and 17 marked with light orange). Sequential serum samples from the patients (baseline, white; weeks 8–12, green; weeks 20–24, magenta; weeks 48–52, blue; >52 weeks, dark orange) and IgG (control) were used to display the intensity distribution of the binding to the peptide on the microarray. The peptide sequences of HER2 ECD and TMD are listed on the left column of the heatmap. HER2 ECD immunogenic domains are marked on the right side of the heatmap: I (residues 42–186; white bar), II (residues 236–363; gray bar), III (residues 324–530; black bar), and IV (residues 531–626; blue stripes). Of note, trastuzumab binds to domain IV and pertuzumab binds to domain II, respectively. Patient samples that showed ≥2.5-fold response over baseline; the samples were marked with blue-lined squares in the rows of corresponding peptides. Patient 17 showed polyclonal responses to numerous peptides throughout HER2 ECD immunogenic domains. Patient 9 showed a ≥2.5-fold increase compared with the baseline reaction against HER2 peptides 11 and 137. The two best responders on this study shared many peptides they had reactions to at baseline or after the vaccination, and many of those appeared to be unique to these two patients only (marked with green dotted lines; peptides 14, 35, 63, 116, 117, 131, 132, and 148). Patient 29 also showed a ≥2.5-fold increase compared with baseline against the peptides in domain IV as patient 9 did, but the responses were not accompanied by the corresponding clinical responses. Nearly all patient samples from all timepoints showed reactions to multiple peptides (marked with black solid lines; peptides 22, 83, 84, 85, and 122) across the previously described immunogenic domains.

Anti-HER2 antibody response after vaccination ≥4-fold over baseline, which was the study-defined positive response, was observed in one patient who had a CR. If ≥2.5-fold over baseline is used as the standard, 3 of 13 patients were positive (**Figure 3** and **Table 4**). Patient 18 had a response that was 2.55-fold increased response against HER2 peptide 138 at week 8 where the antibody response evaluation eligibility was only after three doses of the vaccine. Moreover, the result from the following weeks did not support a sustained response. Numerous peptides throughout different domains of HER2 ICD elicited responses in patient 17 who had CR. Both of the top 2 responders (patient 9 and patient 17) had several shared peptides (peptides 14, 35, 63, 116, 117, 131, 132, and 148) that showed increased reaction at baseline and follow-up sera unique to these two patients. Three patients showed increased reaction in consecutive peptides in immunogenic domain 4, which is the trastuzumab-binding domain, but the HER2 peptides to which the sera of individual patients reacted varied (25). Nearly all patient samples from all timepoints showed reactions to multiple peptides (22, 83, 84, 85, and 122) across the known immunogenic domains.

**Table 4:** Antibody response against HER2 using peptide microarray after at least three doses of the vaccine.

Patient	Response	Week 8	Week 12	Week 28	Week 48	Week 52	Week 76	Week 100
6	PD	NA	−	−	NA	NA	NA	NA
9	PR	≥2.5	≥2.5	NA	NA	−	NA	NA
11	SD	−	−	NA	NA	−	NA	NA
12	SD	NA	−	NA	NA	NA	NA	NA
14	PD	−	−	NA	NA	NA	NA	NA
16	PD	−	−	NA	NA	NA	NA	NA
17	CR	NA	NA	−	NA	≥2.5	NA	≥2.5
18	N/A (NED)	≥2.5	NA	−	−	NA	−	NA
21	SD	−	NA	−	NA	−	NA	−
25	SD	−	NA	−	NA	NA	NA	NA
26	PD	NA	−	−	NA	NA	NA	NA
27	PD	−	NA	−	NA	NA	NA	NA
29	PD	−	≥2.5	NA	NA	NA	NA	NA

The specimens were collected prior to vaccine administration on the corresponding study week. For example, week 8 sample was drawn prior to the administration of the third dose of the vaccine. Thus, the first timepoint that reflects the immune response after the third dose is week 12. Among the limited samples (n = 13) available for analysis, reactivity to HER2 peptides that was ≥2.5-fold compared with baseline was detected in three patients (patients 9, 17, and 29), among which two were the two best responders (patient 9 and patient 17). Patient 18 showed ≥2.5 but only from the sample at week 8 without sustained response. If the ratio of reactivity compared to the baseline was <2.5, it was marked as negative (–).

NA, not available.

#### 3.4.2 Anti-HER2 Cellular Response

**Figure 4:**
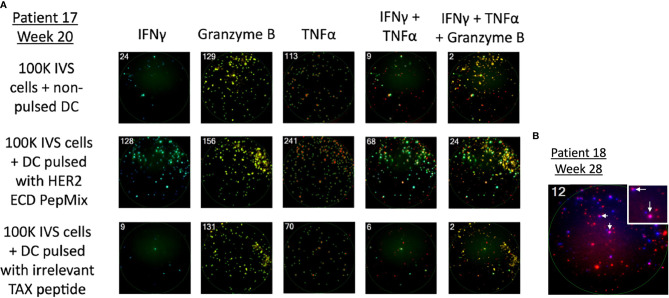
Detection of cellular response against HER2 after vaccination using the FluoroSpot assay. PBMCs were stimulated in the presence of a HER2 peptide mix and measured for the production of IFN-γ, granzyme B, TNF-⍺, and their combinations. **(A)** Representative image of the assay on PBMCs of patient 17 who had complete regression of the lesion after vaccination. The first row is without antigen presentation, the second row is HER2 presented *via* DCs *in vitro*, and the third row shows cells stimulated by an irrelevant peptide. HER2-stimulated cells show a marked increase in spot numbers compared with control rows. **(B)** Representative image of the assay detecting multifunctional lymphocyte response. Taken from patient 18 who received the vaccine as an adjuvant after standard treatment for high-risk muscle-invasive bladder cancer when there was no detectable tumor in imaging studies. The number in the left upper corner indicates positive spots (cells) per well; IFN-γ (blue) and TNF-α (red); dual positive cells (pink) were marked with arrows.

With the exception of IL-4 (*n* = 9), paired pre-vaccination and post-vaccination samples were available for evaluation in 11 patients. Peripheral blood lymphocytes from patients who were stimulated with a HER2 peptide mix showed pre-existing anti-HER2 ECD response when measured for IFN-γ (*n* = 6), granzyme B (*n* = 3), TNF-α (*n* = 5), and IL-4 (*n* = 5 of 9) by the FluoroSpot assay. None of the three patients who had tumor shrinkage that lasted 24 weeks or longer (marked with *) after vaccination showed cellular responses against HER2 ECD in the baseline samples and showed vaccine-induced responses. Among 11 patients who received at least three doses of the vaccine with available paired biospecimens, there was a newly detected production (“induced”) of at least one of IFN-γ, granzyme B, or TNF-α in a total of 10 patients when combining the responses against ECD (patients 9, 12, 17, 20, 21, 26, and 27) and ICD (patients 9, 11, 16, 18, 20, 26, and 27) of HER2 (**Table 5A** and **Figure 4**). Throughout the assay, there was a tendency to have a lower number of patients with responses against the ICD than against the ECD, but there were more post-vaccination responses including newly induced responses than the baseline responses against both domains. Of note, ICD was not included in the transduced antigen (see “epitope spreading” in the *Discussion*). Among 11 patients tested, 8 patients [ECD: patients 9, 12, 17, 25, 26, and 27; ICD: patients 9, 17, 18, 20, 26, and 27; **Table 5B**] were found to have been induced to make multifunctional lymphocyte responses. Several of the patients who showed vaccine-induced responses in **Table 5A** also showed polyfunctional lymphocyte responses to the same domains they reacted to [ECD 9, 12, 26, and 27; ICD 9, 18, 20, 26, and 27, **Table 5B**]. IL-4 production was checked as a surrogate marker of T helper 2 (Th2) cells. Newly induced IL-4 production post-vaccination in cells stimulated by ECD (four patients) and ICD (three patients) HER2 peptide mix was detected (**Table 5C**).

**Table 5A:** Detection of cellular responses against HER2 after vaccination.

Patients		IFNγ				Granzyme B				TNF-α				Summary of post-vaccination anti-HER2 response				
		ECD		ICD		ECD		ICD		ECD		ICD		ECD		ICD		ECD/ICD
		Pre	Post	Pre	Post	Pre	Post	Pre	Post	Pre	Post	Pre	Post	Vaccination response	Induced response only	Vaccination response	Induced response only	Induced response only
18	Adjuvant	+	+	−	−	+	+	−	+	+	+	−	−	+	−	+	+	+
20	Adjuvant	+	+	−	+	+	+	−	−	−	+	−	−	+	+	+	+	+
17	CR*	−	+	−	−	−	+	−	−	−	+	−	−	+	+	−	−	+
9	PR*	−	+	+	+	−	+	−	+	−	+	−	+	+	+	+	+	+
21	SD*	−	−	−	−	−	+	−	−	−	−	−	−	+	+	−	−	+
11	SD	+	−	+	−	−	−	−	+	+	−	−	−	−	−	+	+	+
12	SD	+	−	−	−	−	+	−	−	−	+	−	−	+	+	−	−	+
25	SD	+	+	−	−	−	−	−	−	−	−	−	−	+	−	−	−	−
16	PD	−	−	−	−	+	+	+	−	+	+	−	+	+	−	+	+	+
26	PD	−	+	−	+	−	−	−	+	+	−	+	+	+	+	+	+	+
27	PD	+	+	−	+	−	+	+	+	+	+	+	+	+	+	+	+	+
Total		6	7	2	4	3	8	2	5	5	7	2	4	10	7	7	7	10

**Table 5B:** 

Patients		IFNγ-GB				IFNγ-TNFα				GB-TNFα				IFNγ-GB-TNFα				Summary of post-vaccination anti-HER2 response				
		ECD		ICD		ECD		ICD		ECD		ICD		ECD		ICD		ECD		ICD		ECD/ICD
		Pre	Post	Pre	Post	Pre	Post	Pre	Post	Pre	Post	Pre	Post	Pre	Post	Pre	Post	Vaccination response	Induced response only	Vaccination response	Induced response only	Induced response only
18	Adjuvant	+	+	−	+	+	+	−	+	+	+	−	+	+	+	−	+	+	−	+	+	+
20	Adjuvant	+	+	−	+	+	+	−	+	+	+	−	+	+	+	−	−	+	−	+	+	+
17	CR*	−	+	−	+	+	+	−	+	−	+	+	+	−	+	−	−	+	+	+	+	+
9	PR*	−	+	−	−	−	+	−	+	−	−	−	−	−	−	−	−	+	+	+	+	+
21	SD*	−	−	−	−	−	−	−	−	−	−	−	−	−	−	−	−	−	−	−	−	−
11	SD	+	−	+	−	+	−	+	−	+	−	+	−	+	−	−	−	+	−	+	−	−
12	SD	−	+	−	−	+	+	−	−	+	+	−	−	−	+	−	−	+	+	−	−	+
25	SD	+	+	−	−	+	+	−	−	−	+	−	−	−	−	−	−	+	+	−	−	+
16	PD	+	+	−	−	+	+	−	−	+	+	−	−	+	+	−	−	+	−	−	−	−
26	PD	−	−	−	+	−	+	−	+	−	+	+	+	−	−	−	+	+	+	+	+	+
27	PD	−	+	+	+	−	+	−	+	−	+	+	−	−	+	+	+	+	+	+	+	+
Total		5	8	2	5	7	9	1	6	5	8	4	4	4	6	1	3	10	6	7	6	8

**Table 5C:** 

Patients		IL-4					Summary of post-vaccination anti-HER2 response				
		ECD		ICD			ECD		ICD		ECD/ICD
		Pre	Post	Pre	Post		Vaccination response	Induced response only	Vaccination response	Induced response only	Induced response only
18	Adjuvant	−	+	−	+		+	+	+	+	+
20	Adjuvant	+	+	−	+		+	−	+	+	+
17	CR*	−	+	−	−		+	+	−	−	+
9	PR*	NA	NA	NA	NA		NA	NA	NA	NA	NA
21	SD*	−	−	−	−		−	−	−	−	−
11	SD	+	−	+	+		+	−	+	−	−
12	SD	−	+	−	−		+	+	−	−	+
25	SD	NA	NA	NA	NA		NA	NA	NA	NA	NA
16	PD	+	+	−	−		+	−	−	−	−
26	PD	−	−	−	+		−	−	+	+	+
27	PD	+	+	+	+		+	−	+	−	−
Total		4	6	2	5		7	3	5	3	5

Paired serial specimens were available in 11 patients. The top 2 lines represent the patients who received the vaccine as an adjuvant therapy for high-risk bladder cancer and did not recur during the study period. Patients are listed by the response types. Three patients with an asterisk (*) showed shrinkage of the tumors that lasted 24 weeks or longer when compared with the baseline sum of target lesions. Positive response was defined as the mean of the test specimen being greater than the control mean + 1 standard deviation. As there were baseline positive responses against HER2 as previously reported by other researchers, distinction was made in the right-side post-vaccination response summary columns marked “Induced response only” to denote when the baseline response (“Pre”) was absent and positive response was found only after the vaccination (“Post”) in at least one of the markers or the combinations tested. **(A)** Production of IFN-γ, granzyme B, and TNF-α by PBMCs stimulated by HER2 peptide mix. If there was a response in at least one of IFN-γ, granzyme B, or TNF-α, it was considered as having a positive post-vaccination response. Of the 11 patients tested, 7 developed responses against extracellular domain (ECD) and 7 developed responses against intracellular domain (ICD) of HER2. Combining the response against ECD and ICD, 10 patients (91%) among 11 showed newly induced anti-HER2 response. **(B)** Assessment of multifunctional T-cell response. The detection of simultaneous production in at least two of IFN-γ, granzyme B, or TNF-α in combination was considered a positive response. Of the 11 patients tested, 6 (55%) developed new multifunctional responses against ECD that were not present prior to vaccination and 6 (55%) showed newly detected multifunctional responses against ICD. Of note, ICD was not included in the transduced antigen when the AdHER2 DC vaccine was manufactured. Combining the response against ECD and ICD, 8 patients (72.7%) among 11 showed newly induced anti-HER2 cellular responses. Reviewed by the domains of induced responses, several of the patients with polyfunctional lymphocyte response had vaccine-induced responses in **Table 5A** (ECD 9, 12, 26, and 27; ICD 9, 18, 20, 26, and 27) and in the domains they showed polyfunctional responses. **(C)** Production of IL-4 by peripheral blood lymphocytes after AdHER2 DC vaccination. Production of IL-4 was checked as a surrogate marker of Th2 cell activity. Newly induced IL-4 production after the vaccination in both groups of cells stimulated by ECD and ICD HER2 peptide mix was noted.

GB, granzyme B; ECD, extracellular domain; ICD, intracellular domain; Pre, pre-vaccination; Post, post-vaccination; Adjuvant, bladder cancer adjuvant indication bladder cancer; CR, complete response; PR, partial response; SD, stable disease; PD, progressive disease; NA, not available.

## 4 Discussion

This is the first-in-human clinical trial of an autologous DC vaccine against HER2 in patients with solid tumors. Trastuzumab was a groundbreaking HER2-targeted agent that changed the paradigm of HER2+ breast cancer and laid a foundation for HER2 testing and treatment guidelines. However, targeting a single epitope or binding site by a monoclonal antibody or small molecule poses a risk of losing the target by decreased expression or alteration in the binding site, including point mutations or activation of alternative pathways (26–31). In contrast, a vaccine platform that allows antigen processing by the patient’s own immune system can offer immunologic responses against multiple epitopes, engaging several mechanisms of action. However, the clinical success in using APCs in randomized, controlled clinical trials is limited to sipuleucel-T, the sole therapeutic cancer vaccine that is FDA-approved to date (32–34).

In our analysis, the AdHER2 DC vaccine showed virtually no AEs other than self-limited injection-site reactions and revealed preliminary efficacy as a single agent in patients who had progressed after multiple lines of treatment. The overall safety profile of the total 114 doses was related to the underlying cancer course rather than the vaccine itself. Repeat dosing was not associated with any cumulative or escalated toxicities. Injection-site reactions were self-limited with no systemic responses and resolved in ≤1 week. Cardiac toxicities were carefully monitored based on experience from anti-HER2 monoclonal antibody therapy, and no patients showed impaired cardiac function during the study period for up to 2 years. A study using chimeric antigen receptor (CAR)-T-cell therapy against HER2 reported a fatality associated with interstitial infiltrates in the lungs that led to rapid multiorgan failure that is considered as a cytokine release syndrome in retrospect (35), but no such AEs were observed with the AdHER2 DC vaccine.

A widely held concept in tumor immunology is that larger tumor burdens make the immune system more dysfunctional by promoting suppressive immune modulation and tolerance, either directly by affecting the machinery of tumor cell killing or indirectly by promoting a tumor microenvironment hostile to immune killing (36–38). However, our patients showed responses in both relatively small measurable lesions and advanced, multiple large tumor volumes when the tumor had progressed on standard regimens before enrollment. The absence of clinical benefit at 5 × 10^6^ DL may represent suboptimal antigen stimuli for a meaningful immune response. In DL ≥10 × 10^6^ DCs, 7 of 16 evaluable patients (44%) showed clinical benefit, providing a rationale for further clinical studies of this vaccine. DLs above 10 × 10^6^ did not lead to dose-dependent superior responses. Stable disease falls in the window between not enough progression to meet the criteria for PD and not enough shrinkage to meet the criteria for PR. Slow disease progression with no tumor shrinkage is included as “stable disease” by RECIST definition as long as patients do not show apparent progression with >20% increase in the sum of target lesions.

Patient 17 initially presented with HER2 IHC 3+ cancer but recurred with tumors lacking HER2 expression after vaccination. This suggests a possible immune escape mechanism in response to immunotherapy. Narrowly, immune escape involves antigenic alteration or loss of a specific tumor antigen. More broadly, immune escape is the inherent capacity of cancer to evade immune attack in the face of selective pressure from tumor immunosurveillance (39, 40). This phenomenon has been observed when a target antigen in tumor cells is not essential for tumor survival or fitness and tumor cells can survive by losing the vulnerability. The loss of the target or escape from immunosurveillance has been a challenge. A significant number of patients with HER2+ breast or stomach cancer develop a pre- and post-HER2 status discrepancy thought to be associated with a worse outcome (41–43). In therapeutics with a single target, the risk of developing alterations of the target or drug metabolism impairs the durability of responses (44, 45). Immune responses elicited by dying tumor cells can lead to antigen or epitope spreading, making escape more difficult. However, resistant clones and intratumoral heterogeneity have emerged even after a successful reduction in tumor burden and sometimes even after durable responses (46–48). Determining whether targeting multiple epitopes is a more efficient and durable approach than targeting a fixed epitope by engineering for monoclonal antibodies or effector cells will require further investigation. The disappearance of HER2 expression at the time of progression in patients who initially showed clinical benefit may support the hypothesis that a vaccine-induced immune response exerted immunologic pressure on the original tumor and therefore was effective against that tumor, but was followed by immune escape as a resistance mechanism.

HER2 reporting criteria were originally developed as a guidance in decisions regarding standard-of-care treatment using anti-HER2 monoclonal antibodies in breast cancer, and patients are considered HER2-positive if IHC score is 3+ or FISH shows amplification (49). However, this study included patients whose tumors expressed lower levels of HER2 than the original criteria indicated. In investigational settings, cancers with a HER2 IHC score of 1+ or 2+ with negative FISH are referred to as HER2-low. The rationale for vaccines may be different from monoclonal antibody agents, where lower HER2 expression could have an advantage. Lengthy exposure of the immune system of the patients to an overexpressed antigen, such as HER2 with a IHC score, can lead to immune tolerance, impairing the induction of an effective immune response (50–52). In our study, patient 21 with a score of HER2 IHC 1+ had a 24.8% decrease in the sum of target lesions until a non-target lesion progressed at week 48, suggesting potential benefit from vaccination in patients with HER2-low cancers. From an immunologic standpoint, targeting tumors with lower expression of HER2 may be more promising than using exogenous engineered antibody agents that are designed to target overexpressed tumor antigen. Immunologic targets could initiate an immunologic domino effect, as suggested by the antigen spreading and cross presentation of newly available antigens (53–55). Targeting HER2-low cancer opens treatment options to a new group of patients that represent nearly half of breast and stomach cancers (56).

In analyzing the relationship between clinical laboratory values and response to vaccine, lower total lymphocyte and CD4 T-cell counts were somewhat counterintuitively associated with clinical benefit, but we must be cautious in interpreting the finding given the small size of the study. One of the merits of manipulating DCs for antigen processing using an adenoviral vector is that community-acquired seropositivity against adenovirus will not likely interfere with transduction by neutralizing the viral vector carrying the antigen of interest. In our vaccine platform, the Ad5f35 vector was used to better target DCs and lower the risk of neutralization by pre-existing anti-Ad5 antibodies (19). In our study, seroconversion against adenovirus was noted after vaccination but did not exclude clinical benefit. Immunogenicity assays showed HER2-specific antibody responses in 3 of 13 evaluable patients, including the 2 best responders. Two patients shared many HER2 ECD peptides that showed strong reactions, although interpretation was difficult due to elevated baseline reactivity. Since preclinical studies suggested exclusive antibody-mediated protection, deeper investigation for antibody characterization, including epitope mapping and antibody subtyping, is warranted. Also, the timing of specimen collection could have decreased the sensitivity of antibody detection as all specimens were collected 3 weeks post-vaccination, while the ideal interval from vaccination to antibody measurement is thought to be 10 to 14 days after vaccination when peak immune response typically occurs. Future studies should employ optimal timing of specimen collection which will enable the investigators to better analyze the relationship between clinical response and immunologic response. Identifying the antibody-reactive epitopes of an individual patient could provide new therapeutic targets that could expand treatment options beyond current antibody or small molecule targets (17). Polyclonal antibody responses from a multiepitope vaccine may reduce the risk of immune escape mechanisms. Also, vaccinated individuals may produce their own polyclonal antibodies instead of requiring repeated exogenous monoclonal antibody treatment and may maintain prolonged immune surveillance with the induction of immune memory.

Cellular response data are less clear as the interpretation was hindered by the presence of baseline anti-HER2 immunity, as previously reported in individuals with or without cancer (57). Newly detected lymphocyte responses against HER2 peptides were detected after vaccination in 10 of 11 patients as determined by the production of at least one of IFN-γ, granzyme B, or TNF-⍺. The absence of cellular responses against HER2 ECD at the baseline in all three patients who showed durable tumor shrinkage after vaccination may suggest pre-existing anti-HER2 cellular immunity in other patients that are associated with immune tolerance. Further investigation is warranted to determine if pre-existing anti-HER2 immune responses have any predictive value in the treatment outcome of anti-HER2 vaccines. Also, responses against HER2 ICD peptides that were not part of the vaccine support the concept of antigen spreading of the AdHER2 DC vaccine and its ability to produce multifaceted immune responses compared with engineered targeted therapeutics against a single epitope. While the majority of the patients showed anti-HER2 responses measured by cytokine production, those responses were not exclusive to the patients who showed clinical benefit. Cancer vaccine-induced responses are often assessed by T-cell response, while vaccines against infectious organism are generally considered efficacious when neutralizing antibodies are detected at certain titers regardless of T-cell responses. There have been cancer vaccine studies that support the idea of coordinated humoral and cellular response against the same vaccinated tumor antigen, which means that a vaccine that induced one type of immune response was found to have induced another type of response (58, 59). As the immunogenicity is confirmed, the measures to aid the trafficking of the new effectors to the tumor tissue and to unleash the immune checkpoints to boost immune-mediated tumor cell killing can be considered when developing a combinational strategy as well as the investigation of the changes in the tumor tissues.

Two evaluable patients who had adjuvant treatment for high-risk muscle-invasive bladder cancer after completing standard treatment regimens had no disease recurrence during the study period. This finding has no statistical value but warrants further investigation of the potential role of the vaccine in delaying or preventing recurrence in high-risk individuals with HER2-expressing cancer.

DC vaccine manufacturing is a complex process. Providing cell products with consistent quality requires seamless cell collection, manufacturing, and administration. Potential strategies to improve time-sensitive clinical needs in DC vaccine manufacturing include 1) adoption of an automated, closed cell culture system; 2) batch manufacturing of the cellular product that minimizes human error and variability; and 3) an exploration of allogeneic or artificial APCs or systems which will be pursued to expand the access to the studied vaccine in future trials.

*In vitro* antigen-transduced DCs *in vitro* could bypass inhibitory immune mechanisms in antigen processing in patients. Such inhibitory processes can be present locally in the tumor microenvironment and systemically as humoral components (22, 60). To a significant extent, the restricted benefit of checkpoint inhibitors has been associated with a lack of endogenous immune response in the microenvironment of the so-called cold tumors either due to the absence of antitumor effector T cells or regulatory immune influences that blocks effective trafficking of immune effectors. Thus, combination approaches incorporating a vaccine as a unique tool that can induce effective tumor-specific immune responses such as AdHER2 DC with checkpoint inhibitors and other immune modulatory agents that reduce the influence of inhibitory immune processes have a great potential for synergy.

The AdHER2 DC vaccine investigated in this first-in-human study has demonstrated safety, tolerability, and preliminary antitumor activity as a single agent, setting the stage for the next steps to explore further applications such as combination therapies with checkpoint inhibitors and other immune modulators, as well as neoadjuvant or adjuvant indications for HER2-expressing tumors including HER2-low status.

## Data Availability Statement

The original contributions presented in the study are included in the article/Supplementary Material. Further inquiries can be directed to the corresponding author.

## Ethics Statement

The studies involving human participants were reviewed and approved by the Institutional Review Board, National Institutes of Health. The patients/participants provided their written informed consent to participate in this study.

## Author Contributions

JM, MT, DS, LVW, and JB conceptualized the study. HM, LE, LVW, and BR conducted the clinical trial. HB, DR, VS, SDP, MM, and FB provided clinical correlatives. WW, JM, SP, DS, LVW, and JB contributed to the study drug development and manufacturing. HM, JI, KD, LMW, and JB contributed to the generation of laboratory correlatives. HM, BM, HB, SS, JI, KD, and JB validated and analyzed the data. HM, BM, JI and JB wrote the manuscript. All authors contributed to the article and approved the submitted version.

## Funding

This study was supported by the Intramural Research Program of Center for Cancer Research, National Cancer Institute, National Institutes of Health.

## Conflict of Interest

The authors declare that the research was conducted in the absence of any commercial or financial relationships that could be construed as a potential conflict of interest.

## Publisher’s Note

All claims expressed in this article are solely those of the authors and do not necessarily represent those of their affiliated organizations, or those of the publisher, the editors and the reviewers. Any product that may be evaluated in this article, or claim that may be made by its manufacturer, is not guaranteed or endorsed by the publisher.
